# What Is New and Effective in Treating Refractory Trigeminal Neuralgia?

**DOI:** 10.7759/cureus.73110

**Published:** 2024-11-06

**Authors:** Raquel Branco, Bernardo G Silva, Adriana Pereira, Isabel Amorim, Jorge Jacinto

**Affiliations:** 1 Physical Medicine and Rehabilitation, Hospital Do Divino Espírito Santo, Ponta Delgada, PRT; 2 Physical Medicine and Rehabilitation, Unidade Local De Saúde De Santa Maria, Lisboa, PRT; 3 Physical Medicine and Rehabilitation, Centro De Medicina De Reabilitação De Alcoitão, Lisboa, PRT; 4 Botulinum Toxin Clinic, Gait Analysis Laboratory, Centro De Medicina De Reabilitação De Alcoitão, Lisboa, PRT

**Keywords:** botulinum toxin type a, multiple sclerosis, refractory pain management, refractory trigeminal neuralgia, trigeminal neuralgia

## Abstract

Trigeminal neuralgia (TN) is a severe facial pain disorder characterized by brief, electric shock-like pain triggered by innocuous stimuli, commonly affecting middle-aged women. TN can be classified as classic, secondary, or idiopathic, with the secondary form linked to multiple sclerosis (MS). Treatment typically begins with carbamazepine or oxcarbazepine, but surgical and alternative treatments, including botulinum toxin type A (BoNT-A), may be considered for refractory cases. We present the case of a 47-year-old female with secondary progressive MS and refractory TN, initially diagnosed in 2008. Following a history of failed pharmacological and surgical interventions, including microvascular decompression and gamma knife surgery, the patient was admitted to a rehabilitation center for motor, cognitive, and functional recovery. Her severe pain, which did not respond to conventional medication, impaired her participation in rehabilitation, leading to the use of BoNT-A as a new intervention. A total of 100U of BoNT-A was injected subcutaneously across the most painful facial regions, at 1 cm intervals. Following the BoNT-A injection, the patient reported a marked reduction in pain (VAS score reduced from 8-9/10 to 1/10), less frequent exacerbations, and reduced dependence on emergency analgesics. The only adverse effect observed was transient ipsilateral facial paresis (House-Brackmann grade II). This case underscores the potential of BoNT-A as a valuable adjunctive therapy for TN, particularly in complex patients where traditional medical and surgical options have failed. BoNT-A was well-tolerated, providing substantial pain relief with minimal side effects. Despite the absence of standardized guidelines for its use in TN, this case supports its consideration in refractory cases, highlighting the need for further research to optimize dosing and administration techniques.

## Introduction

Trigeminal neuralgia (TN) is defined by the International Headache Society as "recurrent unilateral brief electric shock-like pain, abrupt in onset and termination, limited to the distribution of one or more divisions of the trigeminal nerve and triggered by innocuous stimuli” [1]. Its incidence ranges from 2.1 to 28.9 per 100,000, with a higher prevalence in women aged 40 and 60 years [2,3]. Three forms of TN are described: classic, secondary, and idiopathic. The classic type, accounting for 75% of cases, is associated with neurovascular compression and morphological changes of the nerve root (atrophy or displacement) demonstrated by MRI or surgery. The secondary type, constituting 15% of cases, is linked to underlying diseases such as multiple sclerosis (MS), space-occupying lesions, arteriovenous malformations, skull-base bone deformities, or genetic causes. The idiopathic type is considered when no apparent cause is found (neither electrophysiological tests nor MRI reveals abnormalities) [1,4]. Most patients experience paroxysmal episodes of unilateral facial pain, often triggered by non-painful stimuli (intra- or extraoral such as light touch, wind, eating, drinking, brushing teeth, shaving, or applying makeup). The pain is described as "stabbing," "electric shock-like," or "shooting," lasting less than two minutes in 75% of cases [1,5]. Continuous, less intense pain between paroxysms is observed in nearly half of the patients, particularly in women, and is less responsive to treatment [6,7]. The second and third divisions of TN are the most affected (69% of all patients), while bilateral TN is rare (1.7%-5%), warranting investigation for secondary causes such as MS [7-10].

Diagnosis is primarily clinical, based on patient history, pain characteristics, and triggers. Neurological examination focuses on trigeminal function and signs of MS or space-occupying lesions. About 30% of patients may exhibit mild hypoesthesia [7]. An MRI of the brain and brainstem is strongly recommended in all patients, along with trigeminal reflex testing, evoked potentials to help the diagnosis, as well as computed tomography to rule out tumors, in cases where MRI is unavailable or contraindicated [10]. Differential diagnosis should consider conditions such as glossopharyngeal neuralgia, persistent idiopathic facial pain, painful trigeminal neuropathy, short-lasting unilateral neuralgiform headache attacks (SUNHA), cluster headache, or odontogenic pathology [8].

The primary treatment for TN involves the use of carbamazepine or oxcarbazepine, which effectively prevents exacerbations in most patients. These medications modulate voltage-gated sodium channels, leading to a decrease in neuronal activity. However, they may cause side effects such as somnolence, dizziness, tremors, and drug interactions, with treatment interruption occurring in nearly 40% of patients [8,11]. Patients on these medications should be monitored for serum sodium levels to detect hyponatremia and undergo an EKG to exclude atrioventricular block. Despite these considerations, carbamazepine (200-1200 mg/day) or oxcarbazepine (300-1800 mg/day) remain the most effective medications, providing initial pain control in almost 90% of patients [10]. In cases of conservative failure or intolerance, it is advisable to switch between carbamazepine and oxcarbazepine or add other drugs like pregabalin, gabapentin, lamotrigine, or baclofen. While these can be used as monotherapy, there is less evidence of efficacy. Topical lidocaine may offer transient relief [12]. Severe exacerbations may require hospital treatment with intravenous drugs such as fosphenytoin or lidocaine, although scientific evidence supporting their efficacy is limited. Opioids are generally not effective in acute exacerbations [8,10,11].

If surgery is indicated, microvascular decompression stands as the first-line treatment, especially in cases with neurovascular compression, showing associations with longer pain relief (61%-88% after five years). Minor complications include sensory loss, transient cranial nerve dysfunction, and aching or burning pain (2% to 7% of cases). Major complications, such as significant cranial nerve dysfunction (2%), stroke (0.3%), and death (0.2%), are less common. Recurrence rates are about 2% annually. Alternative surgical options include procedures damaging the trigeminal ganglion, chemical (glycerol rhizolysis), mechanical (balloon compression), or thermal (radiofrequency thermocoagulation). Stereotactic radiosurgery (‘‘gamma knife’’) targeting the root of the TN has shown symptomatic relief in 33%-56% of patients after five years, with a lower rate of complications compared to microvascular decompression [8,13].

Considering the risks associated with invasive treatments, recent studies suggest the potential positive effect of botulinum toxin type A (BoNT-A) in managing TN. A systematic review reported a 50% decrease in pain severity and frequency when combined with systemic drugs. Another study demonstrated the absence of pain at 14 months post-treatment. However, the dose and method of administration vary widely, and the European Academy of Neurology gives it a “weak recommendation” [10].

## Case presentation

This clinical case details the experience of a 47-year-old female with a history of relapsing-remitting MS since 2000, progressing to secondary progressive MS in 2005. The latest MRI revealed demyelinating lesions across cerebral hemispheres, cerebellum, brainstem, and cervical and dorsal spinal cord (MRI images not provided, information obtained from the respective reports). Currently undergoing targeted Ocrelizumab therapy, her disability, measured by the EDSS (Expanded Disability Status Scale), stands at nine. As such, she was admitted to a rehabilitation center in 2023 with the goal of motor, cognitive, and functional enhancement, which were achieved. However, the emergence of uncontrolled TN during this period presented a significant challenge.

Diagnosed with right TN in 2008, an initial MRI ruled out secondary causes other than MS. Due to severe, refractory pain in 2010, micro decompression of the right Gasser's ganglion was performed using a needle and balloon catheter under fluoroscopic control, providing relief for approximately two years. In 2014, she underwent “gamma knife” surgery, with temporary relief, necessitating the introduction and titration of various medications (anticonvulsant, analgesic, and antidepressant). Upon rehabilitation center admission, the patient was on carbamazepine (400 mg 8/8h), gabapentin (900 mg 8/8h), clomipramine (75 mg 12/12h), and SOS morphine (5 to 10 mg with a 6h interval). She had previously been prescribed oxcarbazepine, amitriptyline, duloxetine, lamotrigine, fentanyl, and tapentadol, all of which were discontinued due to either inadequate efficacy or the occurrence of side effects. The pain, rated 8-9/10 (VAS), impacted her quality of life, functionality, and ability to collaborate in the global rehabilitation program. Significant relief from morphine during emergency situations (SOS) was lacking. Therefore, adjustments were made to the core medication, limited by the suboptimal doses of carbamazepine, which had already been associated with symptomatic hyponatremia requiring correction. In addition to these medication adjustments, topical lidocaine was applied to the areas of heightened pain in the oral mucosa and face.

**Figure 1:**
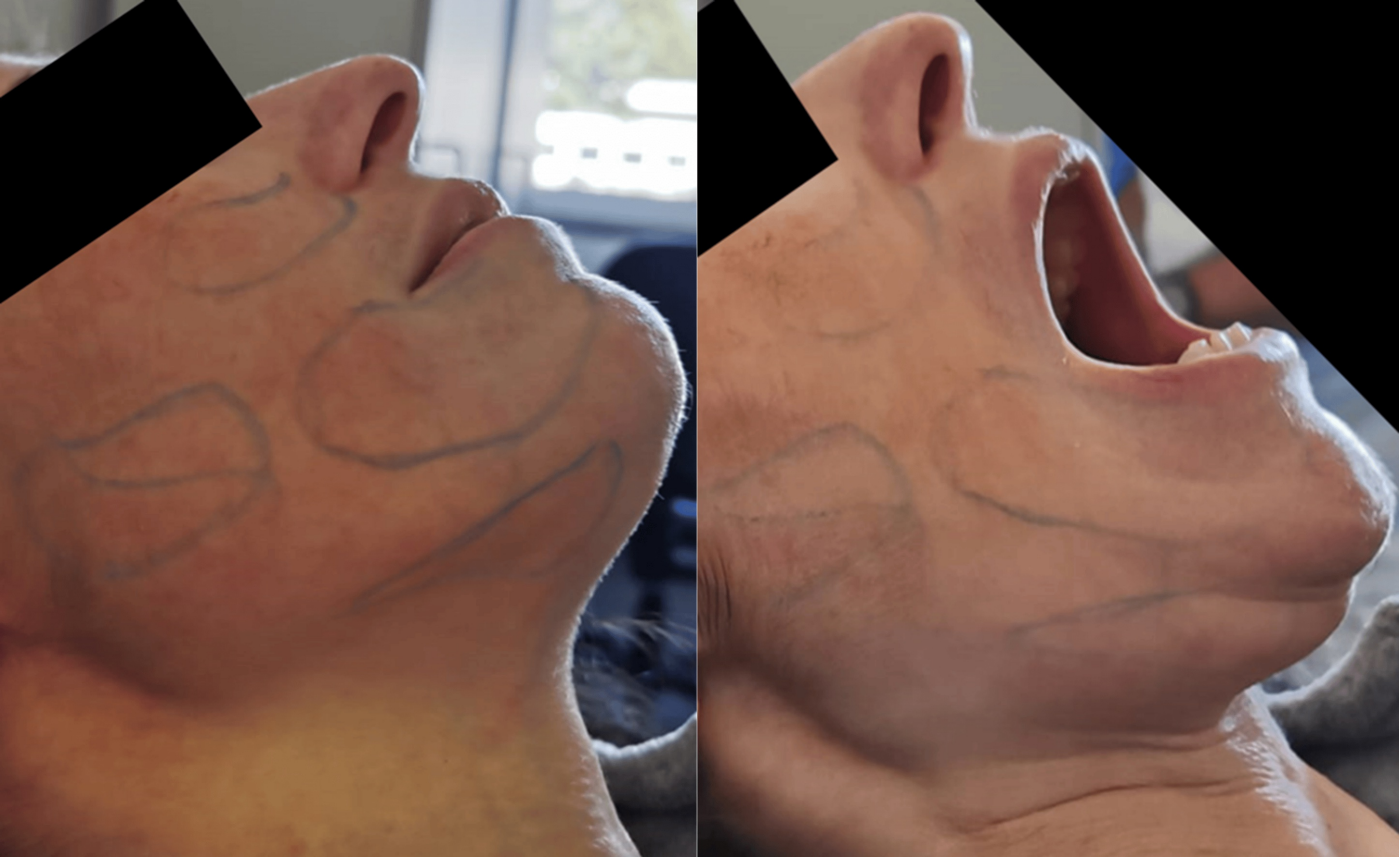
Blue markings outlining the areas of pain that were treated with subcutaneous BoNT-A BoNT-A, botulinum toxin type A

Facing persistent pain, the decision was made to proceed with botulinum toxin treatment, which was administered during the rehabilitation center stay. BoNT-A (Botox®) was subcutaneously injected, with 100U divided strategically among the most painful areas of the face, maintaining approximately 1 cm of distance between each injection site (Figure 1).

After a few days, significant pain relief was observed, resulting in a baseline discomfort level of 1/10 (VAS) and less frequent exacerbations, now rated as 4/10 (VAS). This improvement was accompanied by a reduced reliance on emergency analgesia. The only reported side effect was temporary ipsilateral facial paralysis (House-Brackmann grade II). Upon discharge, pain management included carbamazepine at 400 mg during breakfast and dinner, 200 mg at lunch, gabapentin at 800 mg every eight hours, clomipramine at 75 mg every 12 hours, lidocaine gel at 2% applied in the mouth before brushing teeth, and lidocaine topical at 5% on the painful area of the face at night. This pain relief was observed for four months.

## Discussion

BoNT-A, derived from Clostridium botulinum, functions by impeding acetylcholine release at neuromuscular junctions, thereby inhibiting muscle contraction. Beyond its muscle-relaxing properties, BoNT-A has demonstrated an increasing role in pain reduction, impacting both peripheral and central sensitization. The mechanism underlying the reduction of sensitization by BoNT-A is not fully elucidated, but current understanding suggests its association with the inhibition of neurotransmitter release from peripheral sensory nerves and inflammatory mediators. Notably, substances such as glutamate, substance P, and calcitonin gene-related peptides play crucial roles in sensitization processes. BoNT-A's action extends beyond the periphery, as it seems to centrally block neurotransmission and modulate central opioidergic transmission [4].

This multifaceted impact on neurotransmitter release and modulation of inflammatory mediators contributes to the overall efficacy of BoNT-A in managing conditions like TN. As research progresses, a more detailed understanding of the precise molecular and cellular events involved in BoNT-A's therapeutic effects may emerge, further refining our knowledge of its mechanisms of action. Numerous studies have highlighted the efficacy of BoNT-A in alleviating TN symptoms, showcasing both short-term and long-term pain relief. The benefits are typically observed within six weeks to three months after injection, with sustained relief reported up to 28 months in certain cases. Factors influencing the duration of improvement include patient sex, disease duration, and injected doses exceeding 70U [14,15].

Despite its therapeutic potential, there is a lack of standardized guidelines regarding optimal BoNT-A doses, frequency, and injection techniques. Most literature suggests subcutaneous administration with doses ranging from 25 to 100U, spaced 1 cm apart based on pain distribution [9]. The case discussed involves TN secondary to MS, where initial pharmacological and surgical interventions provided only temporary relief. With classical preventive medications causing hyponatremia, the patient was proposed and accepted BoNT-A therapy, yielding favorable results for four months.

BoNT-A treatment, while generally well-tolerated, may present transient adverse effects. Localized pain, hematoma, edema, asymmetry of the face, and impaired mastication and/or swallowing are among the reported side effects. Nevertheless, these effects tend to be temporary and resolve spontaneously [16]. Facial paralysis, although observed in approximately 14% of cases, was well-received by the patient in this particular instance, considering the substantial relief from persistent pain experienced for many years. Exploring intraoral injections has shown promise in minimizing facial asymmetry, providing relief not only in the oral cavity but also extending to extraoral areas. A case report highlighted the successful treatment of TN with an extraoral trigger zone using intraoral injections of BoNT-A [17].

However, it is important to disclose that this is for now an off-label use and that further high-quality studies are essential to establish conclusive recommendations regarding the optimal use of BoNT-A as a primary therapeutic approach. Key areas for exploration include determining appropriate doses, injection techniques (subcutaneous, intradermal, or submucosal), and overall guidelines for application. Until more robust evidence is available, the consensus among experts leans toward considering BoNT-A when medical or surgical interventions have proven ineffective.

## Conclusions

The case hereby reported illustrates the successful integration of various therapeutic modalities, including surgical interventions, pharmacological agents, and innovative approaches like BoNT-A, in effectively managing TN in a complex MS patient. More studies are needed to establish guidelines for the use of botulinum toxin in these cases, but careful periodic monitoring and interdisciplinary collaboration remain pivotal for sustaining the positive outcomes.
